# A cross-sectional analysis of the association between sleep duration and osteoporosis risk in adults using 2005–2010 NHANES

**DOI:** 10.1038/s41598-021-88739-x

**Published:** 2021-04-27

**Authors:** Chia-Lin Lee, Huey-En Tzeng, Wei-Ju Liu, Chun-Hao Tsai

**Affiliations:** 1 Division of Endocrinology and Metabolism, Department of Internal Medicine, Taichung Veterans General Hospital, Taichung, Taiwan; 2 Department of Public Health, College of Public Health, China Medical University, Taichung, Taiwan; 3 Department of Medical Research, Taichung Veterans General Hospital, Taichung, Taiwan; 4 School of Medicine, National Yang Ming Chiao Tung University, Taipei, Taiwan; 5 Department of Internal Medicine, School of Medicine, Taipei Medical University, Taipei, Taiwan; 6 Graduate Institute of Cancer Biology and Drug Discovery, College of Medical Science and Technology, Taipei Medical University, Taipei, Taiwan, ROC; 7 Department of Internal Medicine, Division of Hematology/Oncology, Taipei Medical University Hospital, Taipei, Taiwan; 8 Department of Orthopedics, China Medical University Hospital, Taichung, Taiwan; 9 School of Medicine, China Medical University, #91 Hsueh-Shih Road, Taichung, 404 Taiwan; 10 Department of Sports Medicine, Sport Medicine, College of Healthcare, China Medical University, Taichung, Taiwan

**Keywords:** Risk factors, Endocrinology, Endocrine system and metabolic diseases, Metabolic bone disease, Epidemiology

## Abstract

Controversy remains regarding the relationship between bone health and sleep. In the literature, the effect of sleep on bone density in the clinical setting varies depending on the definition of normal sleep duration, sleep quality, selected population, and diagnostic tools for bone density. The aim of this study was to examine the association between bone mineral density (BMD)assessed by dual-energy X-ray absorptiometry and sleep duration/quality in the defined adult population from the National Health and Nutrition Examination Survey (NHANES) (a national household survey) within a 6-year period (2005–2010) and explore age differences. The basic variables, metabolic diseases, and bone density in the femoral neck as determined through dual-energy X-ray absorptiometry, were segregated, and analyzed according to different sleep durations (1–4, 5–6,7–8, and > 9 h/day) and sleep quality using multinomial regression models. A total of 12,793 subjects were analyzed. Our results reveal that women aged > 50 years with sleep duration < 5 h/day had a 7.35 (CI 3.438–15.715) odds of osteoporosis than those in other groups. This analysis is based on a nationally representative sample using survey and inspection data and clarifies the relationship between bone density and the effect of the combination of sleep quality and duration.

## Introduction

It is estimated that at least 50% of adults experience significant sleep disturbance, especially elderly individuals^1^. Currently, there is controversy regarding the relationship between bone health and sleep. In the literature, conclusions about the effect of sleep on bone density in a clinical setting vary depending on the definition of normal sleep duration, sleep quality, selected population, and diagnostic tools for bone density. Both long^2–10^ or short^4, 5, 7, 8, 11–13^ self-reported sleep duration have been associated with low bone mineral density (BMD)/osteoporosis or fracture in the literature. Some, studies have not reported an association between sleep duration and BMD^14, 15^. However, in these studies, the diagnostic methods used for osteoporosis varied considerably, including self-reported osteoporosis fracture, BMD by ultrasonic bone densitometry, peripheral quantitative computed tomography, or dual-energy X-ray absorptiometry (DXA)^2–10^. The gold-standard technique for the diagnosis of osteoporosis is based on BMD at either lumbar spine or hip by DXA technique^16^. The controversy regarding the effect of sleep on bone density is also based on sample sizes; most of these studies were cross-sectional community-based^2–9, 11–13^, and only few were study population-based from the National Health and Nutrition Examination Survey (NHANES) dataset, which was 4-year aggregated analysis^7^. Therefore, we would like to expand on the previous NHANES study by 2005–2010 cycle data.

Hip fracture has the worst consequences of patients with osteoporosis^17^ Hip fractures are classified into femoral neck and trochanteric fractures, each having different etiologies^18^. In this study, we specifically focusing on the hip area to measure bone density, as hip fracture is the most adverse of the fragility fractures.

The purpose of the study was to examine the association between BMD using the DXA technique and sleep duration/quality in a defined adult population from extended NHANES data (a national household survey) within 6-year period (2005–2010) and explore age differences.

## Subjects and methods

### Study population and data collection

NHANES is one of a series of health-related programs conducted by the National Center for Health Statistics of the Centers for Disease Control and Prevention, and the database is released periodically. NHANES is a series of cross-sectional national surveys used to examine the health and nutritional status of non-institutionalized Americans. These surveys use stratified multi-stage sampling techniques and documented designs and methods^19^.

**Figure 1:**
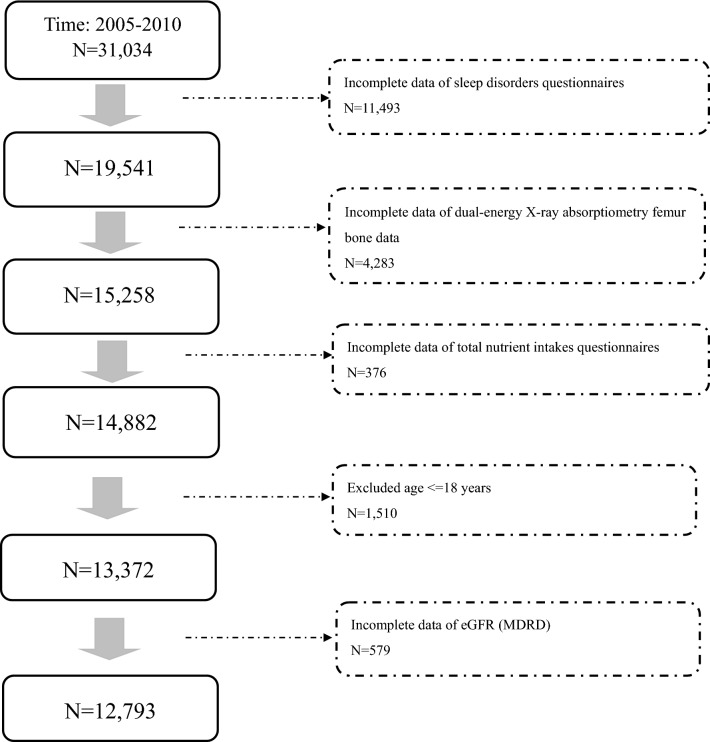
The selection process of subjects from the 2005–2010 NHANES database.

Because the NHANES consists of de-identified secondary data released to the public for research purposes, the NCHS Research Ethics Review Committee approved our investigational procedures, and all subjects or agents provided written informed consent. The study followed relevant guidelines and regulations. The encrypting procedure is consistent so that linkage of claims belonging to the same patient is feasible within the NHANES. The content of examinations includes anthropometrics, health and nutrition questionnaires, and laboratory tests. All subjects completed home interviews. Subjects aged < 18 years and those with incomplete anthropometric data, questionnaires, or laboratory tests were excluded from the study. We analyzed the subjects recorded in NHANES from 2005 to 2010. Figure 1 shows the flow chart for the selection of the study population.

### Definition of sleep duration and quality

The duration of sleep was captured by a single question in NHANES: How much sleep do you usually get at night on weekdays or workdays? ” The response categories range 1–12, with 12 indicating that the subject slept for ≥ 12 h. Sleep duration was analyzed as both a continuous and categorical variable. Based on previous studies^20–22^, categories were assigned toa number of different sleep durations (“very short”: 1–4 h/day;“short”:5–6 h/day;“average”:7–8 h/day; and “long”: > 9 h/day). Sleep quality (yes versus no) was defined by the following questions: “Ever told doctor had trouble sleeping?” and” Ever told by doctor have sleep disorder?”.

### Definition of osteoporosis and age criteria

The study subjects were examined using DXA for BMD (g/cm^2^). BMD of the femoral neck, trochanteric, intertrochanteric, and total femoral areas were measured by a DXA scan (Hologic, Bedford, MA, USA). Quality control was routinely conducted on all DXA machines. We classify the bone health status into low BMD (osteopenia)/osteoporosis/ normal by WHO criteria, which bone mineral density at the femoral neck equal to or less than 2.5 standard deviations below the mean for a young person of the same sex is diagnostic of osteoporosis. Low BMD (or osteopenia) is reported as a T score <  − 1.0 and >  − 2.5^23^.

Bone loss accelerates with aging, especially in menopausal women; 40% of US White women and 13% of US White men aged > 50 years will experience at least one clinically apparent fragility fracture in their lifetime^24^. Therefore, we set 50yearsas the age division for analysis.

The present study was approved by the Human Research Review Committee of the Taichung Veterans General Hospital, Taiwan (CE19051B).

### Statistical analysis

Unless otherwise stated, the data are expressed as the mean ±  ± 95% confidence interval. All reported p-values are bidirectionally < 0.05 denoted statistical significance. Because the survey design of the NHANES study is complex (e.g., complex surveys designed with stratification, clustering, and/or unequal weights), the usual estimates are not appropriate, and all analyses were appropriately weighted to represent the US population. Weighted data were calculated according to analytical guidelines (US National Health and Nutrition Survey: Analytical Guidelines, 2011–2014 and 2015–2016. Available online)^19^. Analysis of variance was used to examine significant differences in baseline demographics and characteristics across groups with different sleep durations. The sample-weighted analysis of variance test was performed using the SAS SURVEYREG Procedure according to the analysis program’s User's Guide. Multinomial logistic regression was used to estimate the impacts of sleep duration on osteoporosis, low BMD (osteopenia) and normal BMD by using the SURVEYLOGISTIC Procedure. We adjusted for age, energy intake, chronic kidney disease status, and body weight. Odds ratio (OR) and 95% confidence interval from multinomial logistic regression were reported. The data were analyzed using SAS software (version 9.4, 2013; SAS, Cary, NC, USA).

### Ethical approval

This study was approved by the Ethics Committee of Taichung Veterans General Hospital (IRB number: CE19051B).

## Results

Initially, 31,034 subjects were considered. After excluding those who did not meet the criteria, 12,793 subjects were enrolled in this study (Fig. 1).

The medical parameters are shown in Table 1. In our study population, most subjects had a sleep duration of 7–8 h/day (54.2%), which was set as reference. The next most prevalent sleep duration group was 5–6 h/day (33.2%); the duration with the fewest instances was 1–4 h/day (5.6%). On average, men had shorter sleep duration than women (men: 6.8 ± 0.02 h/day; women: 7 ± 0.0 3 h/day). There were no significant differences in sleep duration in terms of age or race. There were 13% of the population with fracture history. Of the included subjects, 25% had sleep disorder.

**Table 1:** Characteristics by Sleep duration group.

Variables	Overall	Sleeping hours per day				P-value
		1–4	5–6	7–8	> 9	
	N = 12,739	n = 714	n = 4224	n = 6907	n = 948	
**Age, year**						
Mean (95% CI)	46.25 (45.57, 46.94)	46.43 (44.89, 47.97)	45.81 (45.08, 46.54)	46.33 (45.6, 47.06)	47.51 (45.36, 49.66)	< 0.0001
Male, n (%)	6623 (50)	381 (52)	2251 (54)	3547 (49)	444 (40)	< 0.0001
**Race, n (%)**						
Mexican American	2447 (8)	95 (7)	760 (8)	1428 (9)	164 (8)	< 0.0001
Other Hispanic	1123 (5)	71 (6)	400 (5)	579 (4)	73 (5)	
Non-Hispanic White	6295 (71)	293 (60)	1807 (66)	3674 (75)	521 (74)	
Non-Hispanic Black	2367 (10)	218 (20)	1042 (14)	951 (7)	156 (9)	
Other Race—Including Multi-Racial	561 (6)	37 (7)	215 (7)	275 (5)	34 (5)	
Body mass index, kg/m^2^	27.81 (27.62, 28.01)	28.63 (28.05, 29.22)	28.32 (28.1, 28.55)	27.55 (27.3, 27.79)	27.12 (26.54, 27.71)	< 0.0001
Systolic blood pressure, mm Hg	122.08 (121.53, 122.63)	123.11 (121.51, 124.7)	122.37 (121.65, 123.09)	121.83 (121.17, 122.48)	122.19 (120.76, 123.63)	< 0.0001
Diastolic blood pressure, mm Hg	70.57 (70.06, 71.09)	70.59 (69.5, 71.69)	71.28 (70.63, 71.93)	70.45 (69.9, 71)	68.26 (66.92, 69.59)	< 0.0001
CKD, n (%)	420 (2)	37 (3)	122 (2)	207 (2)	54 (4)	< 0.0001
Diabetes, n (%)	1992 (12)	145 (17)	678 (12)	1000 (11)	169 (13)	< 0.0009
Total cholesterol, mg/dl	197.09 (196.02, 198.15)	198.47 (193.57, 203.36)	196.1 (194.71, 197.49)	197.69 (196.37, 199.02)	195.54 (192.44, 198.64)	< 0.0001
HDL cholesterol, mg/dl	53.31 (52.8, 53.83)	51.73 (49.8, 53.66)	52.49 (51.87, 53.1)	53.77 (53.13, 54.4)	54.41 (52.95, 55.88)	< 0.0001
Triglycerides, mg/dl	155.03 (151.97, 158.08)	165.16 (154.82, 175.49)	156.1 (150.64, 161.56)	154.39 (150.02, 158.76)	148.33 (139.98, 156.68)	< 0.0001
Fasting plasma glucose, mg/dl	97.19 (96.38, 98.01)	99.37 (96.17, 102.57)	97.15 (96.04, 98.26)	96.91 (95.94, 97.88)	98.28 (95.89, 100.66)	< 0.0001
HbA1c, %	5.52 (5.49, 5.55)	5.65 (5.56, 5.74)	5.56 (5.52, 5.59)	5.49 (5.46, 5.52)	5.52 (5.45, 5.59)	< 0.0001
eGFR, mL/min/1.73 m^2^	94.81 (93.77, 95.85)	96.49 (94.88, 98.11)	95.83 (94.55, 97.1)	94.33 (93.29, 95.38)	92.93 (90.37, 95.49)	< 0.0001
Protein intake per day, g/kg	1.09 (1.07, 1.11)	1.01 (0.94, 1.08)	1.08 (1.05, 1.11)	1.11 (1.09, 1.12)	1.05 (1, 1.1)	< 0.0001
Calorie intake, kcal/day/kg	28.55 (28.14, 28.96)	27.37 (25.67, 29.07)	28.71 (28.12, 29.3)	28.61 (28.14, 29.08)	28.12 (26.95, 29.28)	< 0.0001
Calorie intake, kcal/day	2212.02 (2180.94, 2243.1)	2145.07 (2017.73, 2272.4)	2268.75 (2227.56, 2309.93)	2201.88 (2165.83, 2237.92)	2079.89 (1993.21, 2166.56)	< 0.0001
% from carbohydrate	49.7 (49.3, 50)	50.7 (49.6, 51.7)	49.9 (49.5, 50.4)	49.4 (49, 49.8)	49.9 (49.1, 50.8)	< 0.0001
% from fat	34.3 (34, 34.6)	33.8 (32.9, 34.6)	34.3 (33.9, 34.6)	34.3 (34, 34.7)	34.2 (33.5, 34.9)	< 0.0001
% from protein	16 (15.9, 16.2)	15.6 (15.1, 16)	15.8 (15.6, 16)	16.2 (16, 16.4)	15.8 (15.5, 16.2)	< 0.0001
Femoral neck BMD	0.84 (0.835, 0.844)	0.827 (0.814, 0.841)	0.85 (0.842, 0.857)	0.837 (0.832, 0.843)	0.821 (0.808, 0.835)	< 0.0001
Femoral neck t score	− 0.435 (− 0.472, − 0.398)	− 0.542 (− 0.643, − 0.441)	− 0.374 (− 0.43, − 0.319)	− 0.448 (− 0.488, − 0.408)	− 0.533 (− 0.633, − 0.433)	< 0.0001
Trochanter BMD	0.737 (0.733, 0.74)	0.718 (0.705, 0.731)	0.746 (0.74, 0.751)	0.735 (0.731, 0.74)	0.715 (0.705, 0.726)	< 0.0001
Trochanter t score	− 0.092 (− 0.126, − 0.059)	− 0.277 (− 0.388, − 0.167)	− 0.029 (− 0.078, 0.019)	− 0.096 (− 0.131, − 0.061)	− 0.229 (− 0.316, − 0.141)	< .0001
Intertrochanter BMD	1.15 (1.145, 1.156)	1.14 (1.122, 1.158)	1.162 (1.153, 1.171)	1.149 (1.143, 1.155)	1.114 (1.1, 1.128)	< 0.0001
Intertrochanter t score	− 0.005 (− 0.04, 0.03)	− 0.088 (− 0.196, 0.02)	0.042 (− 0.011, 0.095)	− 0.004 (− 0.042, 0.033)	− 0.167 (− 0.25, − 0.085)	< 0.0001
Total femur BMD	0.976 (0.971, 0.981)	0.962 (0.947, 0.977)	0.986 (0.979, 0.994)	0.974 (0.969, 0.98)	0.947 (0.935, 0.959)	< 0.0001
Total femur t score	− 0.116 (− 0.152, − 0.081)	− 0.234 (− 0.34, − 0.128)	− 0.062 (− 0.115, − 0.01)	− 0.119 (− 0.156, − 0.081)	− 0.265 (− 0.35, − 0.18)	< 0.0001
Sleep disorder( +)	3035 (25)	385 (55)	1239 (31)	1215 (20)	196 (24)	< 0.0001
**Broken or fractured, n (%)**						
Hip	153 (1)	10 (2)	60 (1)	65 (1)	18 (2)	0.0056
Wrist	1148 (10)	94 (16)	387 (11)	571 (9)	96 (12)	0.0003
Spine	271 (2)	38 (6)	84 (2)	129 (2)	20 (3)	< 0.0001

Subjects who had 1–4 h ‘sleep time were predominantly male, younger, and had higher body mass index (all p < 0.001). They also had higher levels of fasting glucose, hemoglobin A1c, total cholesterol, triglycerides, systolic blood pressure, and diastolic blood pressure; however, they had lower levels of high-density lipoprotein (all p < 0.001). There were more subjects with diabetes mellitus in this group compared with the other sleep duration groups. In this group, 55% had sleep disorder.

BMD (T-score) over femoral neck, trochanteric, intertrochanteric, and total femoral areas in 4 type of sleep duration were shown in Table 2. We used FN BMD to calculated T-scores, and classified participants into 4 type of sleep duration. The femur neck, as the primary site for osteoporosis diagnosis, we further reclassify the cases into low BMD (osteopenia) /osteoporosis/ normal by WHO criteria. The classification based other femur sites was shown in the supplemental material (please see the Supplementary Tables S1–S3). Sleep duration was significantly associated with diagnosis of osteoporosis. While the impact of sleep on the occurrence of osteoporosis based on the WHO definition, there was a higher risk of osteoporosis or low bone density in the case of sleep for less than 4 h (Table 3). This phenomenon is especially obvious in women (Osteoporosis vs. Normal-(OR 4.082 (CI 2.107–7.91); Low BMD vs. Normal- OR 1.753 (CI 1.238–2.483)) and people over 50-year-old (Osteoporosis vs. Normal-OR 3.197 (CI 1.808–5.655); Low BMD vs. Normal-OR 1.709 (CI 1.216–2.403)). The quality of sleep did not affect the bone density statistically significant.

**Table 2:** BMD (T-score) over femoral neck, trochanteric, intertrochanteric, and total femoral areas in 4 type of sleep duration.

Region of interest		Sleeping hours per day				P for trend
		1–4	5–6	7–8	> 9	
**Femoral neck**						
Overall	BMD	0.815 (0.792, 0.837)	0.833 (0.814, 0.852)	0.831 (0.813, 0.849)	0.831 (0.815, 0.846)	0.3459
	T score	− 0.636 (− 0.757, − 0.514)	− 0.49 (− 0.572, − 0.407)	− 0.502 (− 0.582, − 0.422)	− 0.511 (− 0.595, − 0.427)	0.3031
Age < 50	BMD	0.88 (0.858, 0.902)	0.89 (0.871, 0.909)	0.886 (0.869, 0.904)	0.887 (0.868, 0.905)	0.8095
	T score	− 0.106 (− 0.278, 0.066)	− 0.032 (− 0.183, 0.12)	− 0.058 (− 0.196, 0.079)	− 0.061 (− 0.205, 0.082)	0.8134
Age ≥ 50	BMD	0.731 (0.715, 0.747)	0.762 (0.755, 0.769)	0.762 (0.755, 0.768)	0.753 (0.739, 0.767)	0.1559
	T score	− 1.299 (− 1.422, − 1.175)	− 1.046 (− 1.102, − 0.99)	− 1.045 (− 1.094, − 0.996)	− 1.117 (− 1.229, − 1.006)	0.1215
Male	BMD	0.851 (0.836, 0.866)	0.866 (0.857, 0.874)	0.857 (0.85, 0.864)	0.872 (0.854, 0.89)	0.9096
	T score	− 0.578 (− 0.69, − 0.466)	− 0.47 (− 0.534, − 0.406)	− 0.53 (− 0.581, − 0.479)	− 0.426 (− 0.559, − 0.294)	0.9096
Female	BMD	0.778 (0.762, 0.794)	0.8 (0.791, 0.809)	0.804 (0.797, 0.812)	0.793 (0.78, 0.807)	0.0881
	T score	− 0.687 (− 0.82, − 0.554)	− 0.502 (− 0.574, − 0.429)	− 0.463 (− 0.525, − 0.402)	− 0.556 (− 0.67, − 0.443)	0.0881
Sleep disorder (− )	BMD	0.838 (0.823, 0.853)	0.839 (0.831, 0.847)	0.837 (0.83, 0.844)	0.836 (0.825, 0.848)	0.4686
	T score	− 0.455 (− 0.573, − 0.338)	− 0.442 (− 0.504, − 0.38)	− 0.457 (− 0.509, − 0.404)	− 0.468 (− 0.557, − 0.379)	0.5152
Sleep disorder ( +)	BMD	0.79 (0.773, 0.806)	0.816 (0.806, 0.826)	0.809 (0.797, 0.821)	0.811 (0.783, 0.839)	0.4362
	T score	− 0.829 (− 0.96, − 0.697)	− 0.621 (− 0.702, − 0.539)	− 0.674 (− 0.768, − 0.581)	− 0.665 (− 0.896, − 0.434)	0.4428
**Trochanter**						
Overall	BMD	0.704 (0.686, 0.722)	0.729 (0.712, 0.746)	0.728 (0.712, 0.744)	0.724 (0.709, 0.739)	0.0491
	T score	− 0.399 (− 0.515, − 0.283)	− 0.169 (− 0.245, − 0.092)	− 0.17 (− 0.246, − 0.093)	− 0.214 (− 0.312, − 0.116)	0.0356
Age < 50	BMD	0.732 (0.707, 0.757)	0.753 (0.731, 0.776)	0.748 (0.725, 0.77)	0.746 (0.722, 0.769)	0.8866
	T score	− 0.113 (− 0.34, 0.113)	0.083 (− 0.124, 0.291)	0.035 (− 0.169, 0.24)	0.008 (− 0.204, 0.219)	0.9542
Age ≥ 50	BMD	0.664 (0.649, 0.68)	0.695 (0.688, 0.702)	0.701 (0.696, 0.706)	0.692 (0.678, 0.706)	0.0014
	T score	− 0.781 (− 0.923, − 0.639)	− 0.493 (− 0.554, − 0.432)	− 0.432 (− 0.48, − 0.385)	− 0.514 (− 0.644, − 0.384)	0.0007
Male	BMD	0.759 (0.743, 0.775)	0.781 (0.772, 0.79)	0.774 (0.767, 0.782)	0.779 (0.762, 0.796)	0.8942
	T score	− 0.178 (− 0.315, − 0.041)	0.009 (− 0.07, 0.088)	− 0.049 (− 0.114, 0.017)	− 0.009 (− 0.151, 0.132)	0.8942
Female	BMD	0.652 (0.637, 0.668)	0.679 (0.672, 0.685)	0.685 (0.68, 0.69)	0.674 (0.662, 0.686)	0.0140
	T score	− 0.584 (− 0.74, − 0.429)	− 0.316 (− 0.383, − 0.249)	− 0.256 (− 0.309, − 0.203)	− 0.365 (− 0.488, − 0.242)	0.0140
Sleep disorder (-)	BMD	0.718 (0.704, 0.733)	0.734 (0.727, 0.741)	0.73 (0.724, 0.736)	0.728 (0.716, 0.74)	0.4942
	T score	− 0.266 (− 0.401, − 0.131)	− 0.124 (− 0.193, − 0.054)	− 0.157 (− 0.213, − 0.102)	− 0.178 (− 0.287, − 0.068)	0.6214
Sleep disorder ( +)	BMD	0.694 (0.678, 0.709)	0.717 (0.708, 0.726)	0.722 (0.712, 0.733)	0.711 (0.687, 0.735)	0.0317
	T score	− 0.497 (− 0.641, − 0.354)	− 0.271 (− 0.354, − 0.188)	− 0.229 (− 0.328, − 0.129)	− 0.344 (− 0.581, − 0.107)	0.0405
**Intertrochanter**						
Overall	BMD	1.118 (1.1, 1.135)	1.136 (1.117, 1.155)	1.138 (1.121, 1.154)	1.126 (1.108, 1.143)	0.3646
	T score	− 0.218 (− 0.317, − 0.118)	− 0.102 (− 0.173, − 0.031)	− 0.086 (− 0.152, − 0.021)	− 0.171 (− 0.256, − 0.086)	0.3025
Age < 50	BMD	1.174 (1.141, 1.207)	1.184 (1.152, 1.217)	1.181 (1.149, 1.212)	1.178 (1.148, 1.207)	0.6906
	T score	0.171 (− 0.036, 0.377)	0.232 (0.027, 0.437)	0.215 (0.016, 0.415)	0.182 (− 0.003, 0.368)	0.7028
Age ≥ 50	BMD	1.048 (1.027, 1.068)	1.079 (1.069, 1.089)	1.088 (1.08, 1.097)	1.067 (1.05, 1.084)	0.0249
	T score	− 0.681 (− 0.808, − 0.553)	− 0.472 (− 0.537, − 0.407)	− 0.409 (− 0.466, − 0.353)	− 0.548 (− 0.659, − 0.436)	0.0166
Male	BMD	1.186 (1.169, 1.203)	1.204 (1.193, 1.216)	1.197 (1.189, 1.206)	1.199 (1.175, 1.223)	0.7633
	T score	− 0.14 (− 0.239, − 0.04)	− 0.032 (− 0.097, 0.032)	− 0.073 (− 0.124, − 0.022)	− 0.064 (− 0.206, 0.078)	0.7633
Female	BMD	1.052 (1.033, 1.07)	1.068 (1.059, 1.078)	1.079 (1.071, 1.088)	1.057 (1.042, 1.072)	0.1287
	T score	− 0.269 (− 0.399, − 0.14)	− 0.152 (− 0.222, − 0.082)	− 0.076 (− 0.136, − 0.015)	− 0.232 (− 0.339, − 0.124)	0.1287
Sleep disorder (−)	BMD	1.144 (1.125, 1.163)	1.143 (1.132, 1.153)	1.142 (1.134, 1.15)	1.129 (1.114, 1.143)	0.1344
	T score	− 0.055 (− 0.178, 0.067)	− 0.061 (− 0.131, 0.008)	− 0.06 (− 0.112, − 0.009)	− 0.149 (− 0.24, − 0.059)	0.1790
Sleep disorder ( +)	BMD	1.095 (1.075, 1.115)	1.12 (1.108, 1.132)	1.122 (1.107, 1.138)	1.113 (1.079, 1.147)	0.1278
	T score	− 0.363 (− 0.49, − 0.235)	− 0.201 (− 0.279, − 0.122)	− 0.184 (− 0.283, − 0.084)	− 0.253 (− 0.489, − 0.018)	0.1437
**Total femur**						
Overall	BMD	0.944 (0.925, 0.963)	0.964 (0.945, 0.983)	0.965 (0.948, 0.982)	0.958 (0.941, 0.975)	0.1393
	T score	− 0.361 (− 0.464, − 0.259)	− 0.207 (− 0.28, − 0.135)	− 0.197 (− 0.265, − 0.128)	− 0.258 (− 0.344, − 0.173)	0.1083
Age < 50	BMD	0.992 (0.965, 1.02)	1.006 (0.979, 1.032)	1.002 (0.977, 1.028)	1.001 (0.976, 1.026)	0.9652
	T score	0.03 (− 0.175, 0.234)	0.129 (− 0.067, 0.325)	0.108 (− 0.082, 0.299)	0.086 (− 0.1, 0.272)	0.9963
Age ≥ 50	BMD	0.881 (0.864, 0.897)	0.913 (0.905, 0.921)	0.919 (0.913, 0.926)	0.904 (0.89, 0.919)	0.0123
	T score	− 0.848 (− 0.971, − 0.726)	− 0.603 (− 0.664, − 0.542)	− 0.55 (− 0.599, − 0.5)	− 0.665 (− 0.778, − 0.553)	0.0075
Male	BMD	1.005 (0.99, 1.021)	1.024 (1.015, 1.034)	1.018 (1.01, 1.025)	1.023 (1.002, 1.043)	0.9330
	T score	− 0.24 (− 0.346, − 0.134)	− 0.109 (− 0.174, − 0.043)	− 0.157 (− 0.208, − 0.105)	− 0.121 (− 0.261, 0.019)	0.9330
Female	BMD	0.884 (0.869, 0.9)	0.905 (0.897, 0.913)	0.914 (0.907, 0.921)	0.898 (0.885, 0.911)	0.0365
	T score	− 0.456 (− 0.585, − 0.327)	− 0.284 (− 0.351, − 0.218)	− 0.212 (− 0.269, − 0.155)	− 0.347 (− 0.453, − 0.241)	0.0365
Sleep disorder (−)	BMD	0.966 (0.951, 0.981)	0.97 (0.961, 0.979)	0.969 (0.962, 0.975)	0.962 (0.949, 0.974)	0.3198
	T score	− 0.198 (− 0.311, − 0.085)	− 0.165 (− 0.231, − 0.098)	− 0.171 (− 0.222, − 0.119)	− 0.228 (− 0.322, − 0.134)	0.3996
Sleep disorder ( +)	BMD	0.925 (0.908, 0.942)	0.95 (0.941, 0.96)	0.952 (0.939, 0.964)	0.944 (0.915, 0.972)	0.1027
	T score	− 0.505 (− 0.63, − 0.38)	− 0.31 (− 0.383, − 0.237)	− 0.298 (− 0.393, − 0.204)	− 0.368 (− 0.601, − 0.136)	0.1138

Adjust for age, gender, energy, weight, CKD.

*There is significant difference P < 0.05.

**Table 3:** Diagnosis of osteoporosis, low BMD, or normal bone density based on T score over femoral neck.

	Sleeping hours per day							P for trend
	1–4		5–6		7–8	> 9		
	OR 95%CI	P-value	OR 95%CI	P-value	OR 95%CI	OR 95%CI	P-value	
**Overall**								
Osteoporosis vs. Normal	2.489 (1.463, 4.235)	0.0008**	1.234 (0.852, 1.787)	0.2656	REF	1.584 (0.995, 2.521)	0.0526	0.3106
Low BMD vs. Normal	1.351 (1.082, 1.686)	0.0078**	1.009 (0.894, 1.139)	0.8817	REF	0.94 (0.739, 1.197)	0.6163	0.1236
Osteoporosis vs. Low BMD	1.843 (1.114, 3.048)	0.0173*	1.223 (0.874, 1.711)	0.2405	REF	1.684 (1.059, 2.678)	0.0276	0.6403
**Male**								
Osteoporosis vs. Normal	1.302 (0.46, 3.686)	0.6194	0.971 (0.581, 1.623)	0.911	REF	0.912 (0.445, 1.869)	0.8021	0.8044
Low BMD vs. Normal	1.074 (0.785, 1.469)	0.6568	0.953 (0.812, 1.118)	0.5559	REF	0.791 (0.581, 1.076)	0.1357	0.6324
Osteoporosis vs. Low BMD	1.212 (0.45, 3.265)	0.703	1.019 (0.635, 1.636)	0.9381	REF	1.154 (0.602, 2.211)	0.6665	0.9223
**Female**								
Osteoporosis vs. Normal	4.082 (2.107, 7.91)	< 0.0001**	1.394 (0.926, 2.099)	0.1113	REF	2.119 (1.121, 4.005)	0.0208	0.2878
Low BMD vs. Normal	1.753 (1.238, 2.483)	0.0016**	1.075 (0.904, 1.278)	0.4139	REF	1.096 (0.766, 1.569)	0.6169	0.0643
Osteoporosis vs. Low BMD	2.329 (1.21, 4.481)	0.0114*	1.297 (0.898, 1.874)	0.1654	REF	1.933 (1.075, 3.475)	0.0276	0.6935
**Age < 50**								
Osteoporosis vs. Normal	1.509 (0.146, 15.579)	0.7296	0.912 (0.222, 3.75)	0.8983	REF	2.482 (0.449, 13.709)	0.2972	0.7088
Low BMD vs. Normal	1.085 (0.775, 1.518)	0.6346	1.058 (0.896, 1.249)	0.5066	REF	0.928 (0.637, 1.354)	0.6996	0.4075
Osteoporosis vs. Low BMD	1.391 (0.141, 13.726)	0.7774	0.862 (0.212, 3.505)	0.8356	REF	2.673 (0.464, 15.389)	0.2709	0.6342
**Age ≥ 50**								
Osteoporosis vs. Normal	3.197 (1.808, 5.655)	< .0001**	1.223 (0.84, 1.781)	0.2939	REF	1.639 (1.061, 2.53)	0.0259	0.1901
Low BMD vs. Normal	1.709 (1.216, 2.403)	0.002*	0.958 (0.81, 1.134)	0.6196	REF	1.017 (0.776, 1.334)	0.9009	0.2494
Osteoporosis vs. Low BMD	1.871 (1.096, 3.192)	0.0216*	1.276 (0.904, 1.801)	0.1657	REF	1.611 (1.068, 2.429)	0.023	0.4232
**Sleep disorder (**−**)**								
Osteoporosis vs. Normal	1.978 (0.914, 4.279)	0.0832	1.278 (0.818, 1.998)	0.2819	REF	1.266 (0.874, 1.834)	0.2129	0.2917
Low BMD vs. Normal	1.032 (0.755, 1.412)	0.8425	1.006 (0.893, 1.133)	0.9217	REF	0.911 (0.693, 1.197)	0.5028	0.5805
Osteoporosis vs. Low BMD	1.916 (0.85, 4.322)	0.117	1.27 (0.829, 1.947)	0.2719	REF	1.39 (0.939, 2.058)	0.1003	0.4123
**Sleep disorder (+)**								
Osteoporosis vs. Normal	2.361 (0.922, 6.046)	0.0733	1.069 (0.578, 1.976)	0.8311	REF	2.601 (0.932, 7.255)	0.0678	0.9784
Low BMD vs. Normal	1.56 (1.107, 2.198)	0.011*	0.988 (0.77, 1.269)	0.9276	REF	1.018 (0.577, 1.795)	0.952	0.1865
Osteoporosis vs. Low BMD	1.514 (0.662, 3.463)	0.3263	1.082 (0.618, 1.894)	0.7838	REF	2.556 (1.097, 5.957)	0.0297	0.6005

**There is significant difference P < 0.001.

*There is significant difference P < 0.05.

We further evaluate the combined effect of sleep hours, gender, and age. As shown in Table 4, in the case of females aged over fifty with sleep hours less than 5 h/day, the odds ratio of osteoporosis was 7.35 (CI 3.438–15.715) and low BMD was 3.002 (CI 1.828–4.932), respectively. However, there is no significant difference in diagnosis of osteoporosis by the effect of self-report sleep quality (Table 3/4).

**Table 4:** Effect of sleep hours and sleep disorder in over fifty-year-old female on bone density in femoral neck.

	Sleeping hours per day							P for trend
	1–4		5–6		7–8	> 9		
	OR 95%CI	P-value	OR 95%CI	P-value	OR 95%CI	OR 95%CI	P-value	
**Female & Age > 50**								
Osteoporosis vs. Normal	7.35 (3.438–15.715)	< 0.0001**	1.256 (0.799–1.974)	0.3239	REF	1.97 (1.063–3.65)	0.0311	0.1214
Low BMD vs. normal	3.002 (1.828–4.932)	< 0.0001**	1.037 (0.812–1.324)	0.7696	REF	1.141 (0.747–1.743)	0.5415	0.0301*
Osteoporosis vs. low BMD	2.448 (1.222–4.905)	0.0116*	1.211 (0.838–1.749)	0.3085	REF	1.727 (1.032–2.888)	0.0374	0.5852
**Other**								
Osteoporosis vs. normal	1.276 (0.466–3.492)	0.6355	1.203 (0.733–1.976)	0.4644	REF	1.276 (0.6–2.712)	0.5271	0.7844
Low BMD vs. normal	1.117 (0.843–1.482)	0.4403	1.008 (0.878–1.156)	0.9123	REF	0.852 (0.631–1.15)	0.2951	0.3435
Osteoporosis vs. Low BMD	1.142 (0.432–3.016)	0.7892	1.194 (0.736–1.937)	0.4722	REF	1.498 (0.712–3.149)	0.2867	0.9866
**With sleep disorder**								
Female & Age > 50								
Osteoporosis vs. normal	5.578 (1.603–19.412)	0.0069*	1.06 (0.47–2.39)	0.8879	REF	2.519 (0.725–8.753)	0.1461	0.5878
Low BMD vs. normal	2.738 (1.396–5.37)	0.0034*	0.878 (0.557–1.383)	0.5737	REF	0.906 (0.346–2.372)	0.8411	0.1183
Osteoporosis vs. low BMD	2.037 (0.669–6.201)	0.2102	1.208 (0.618–2.361)	0.5803	REF	2.779 (0.948–8.148)	0.0625	0.8613
Other								
Osteoporosis vs. normal	2.182 (1.126–4.226)	0.0208*	1.295 (0.853–1.966)	0.2248	REF	1.39 (0.876–2.204)	0.1619	0.2619
Low BMD vs. normal	1.219 (0.945–1.574)	0.1276	1.037 (0.917–1.173)	0.5629	REF	0.912 (0.715–1.165)	0.4612	0.1701
Osteoporosis vs. low BMD	1.789 (0.935–3.422)	0.0788	1.249 (0.844–1.848)	0.2665	REF	1.523 (0.941–2.467)	0.0869	0.552

**There is significant difference P < 0.001.

*There is significant difference P < 0.05.

## Discussion

This large population-based study revealed that women aged > 50 years with sleep duration < 5 h/day had odds ratio 7.35 (CI 3.43–15.71) with osteoporosis and subjects with poor sleep quality had 5.57 (CI 1.60–19.41) odds of osteoporosis. We assessed the quality of sleep to identify subjects suffering from sleep disorder in a manner comparable with previous NHANES cohort studies^7, 21^. The analysis showed that sleep duration rather than the sleep quality influence the bone density.

Sleep affects bone metabolism and bone density through multiple mechanisms. It includes alterations in the normal rhythmicity of bone cells, hormone levels (e.g., growth hormones, sex steroids, cortisol), increases in sympathetic tone^13, 25^, inflammation^26^, metabolic derangements^27^, or fatigue/physical inactivity^28^. Previous evidence has shown that sleep architecture varies with age. Total nocturnal sleep time and total sleep time decrease with aging^29^. A decline in sleep quality reduces the chance to reach slow-wave sleep, during which most growth hormones are secreted^30–32^. When the depth of sleep is insufficient, the reduction in growth hormone secretion leads to bone loss^33^.

The defined sleep duration associated with osteoporosis or high risk of fracture differs in the literature; short/insufficient sleep durations were < 5^2, 12^, 6^6, 14, 34^, and 6.5 h/day^11^. Considering that sleep duration decreases with age^30^, age may be a factor affecting BMD. However, the age criteria of assessing a population also vary in the literature, and include > 18 years^8, 12^, 20–66 years^11^, middle-age (> 40 years^4, 5, 35^; 45 years^2, 3, 13, 36^; and 50 years^7, 9, 20^), and the elderly (> 60 years^6, 10, 14, 34^; and 69 years^37^). The sleep pattern also differs between genders^38^. Middle-aged women need longer^29^ and more slow-wave^29, 39^ sleep time than men.

The majority of subjects in previous research studies were women^12, 37, 40^. To clarify the effect of sleep/age/gender in bone health, a large population-based study with a standard measurement such as NHANES is warranted to avoid this type of bias.

By selecting a population in the NHANES (i.e., adults aged > 50 years between 2005–2006 and 2007–2008), Cunningham et al. found that a sleep duration < 6 h per night was associated with a significantly increased risk of osteoporosis in those aged > 65 years^7^. Similarly, using NHANES, the present study analyzed the sleep duration/quality in the whole adult group of both genders for a more comprehensive interpretation of the relationship between sleep and bone density. Women aged > 50 years who had short sleep duration were at 7.35 (CI 3.43–15.71) odds of osteoporosis and 3.002 (CI 1.82–4.93) odds of low bone density compared with men, younger individuals, and those with longer sleep duration.

In conditions of stress and lack of sleep, an increase in systemic inflammation is more dominant in women^41^, and this also contributes to bone loss. Moreover, the lack of estrogen in postmenopausal women can exacerbate bone loss^42, 43^. These findings may explain the lower bone density observed in womenaged50yearswith poor sleep quality and shorter sleep duration in this study.

Consistent with the findings of another study^20^, our results revealed the critical effect of sleep quality on bone density. This observation may explain the significant association of both short and prolonged (> 9 h/day) sleep duration with the risk of osteoporosis^8, 44^. Sleep quality in most elderly individuals is poor. Therefore, we can conclude that early screening and intervention for bone density in elderly patients with insomnia may improve their quality of life.

Most previous studies have not evaluated the combined effects of sleep time and quality; in addition, there are various methods for evaluating sleep quality. In the literature, self-reported sleep is associated with an increased risk of osteoporosis^4^; while using the Pittsburgh Sleep Quality Index (PSQI) to assess sleep quality, the results show that it will cause bone loss^43^. However, the interaction between sleep quality and sleep duration, and comorbidity is complicated. The effect of quality is not so obvious after adjusting these confounding factors^40, 45^.

There were several limitations to our study. Firstly, we did not use the lumbar spine BMD DXA data in the diagnosis of osteoporosis; however, the T-score from hip BMD more reliably reflects the risk of hip fracture^46^. Ochs-Balcomet al. reported a similar pattern for hip and spine BMD by analyzing 11,084 postmenopausal women (aged > 50 years) from the Women’s Health Initiative^40^. Secondly, this was a cross-sectional study, similar to previous population-based studies^2–9, 11–13^, which limits the ability to measure temporality. Hence, causality may not be determined. However, we examined a 6-year period (2005–2006/2007–2008/2009–2010) of the NHANES to avoid bias as much as possible. Thirdly, information regarding sleep was self-reported in our study. Self-reported information is less accurate than objective measurements. The level of disagreement between subjective and objective measurements of sleep duration increased with male gender, poor cognitive function, and functional disability, particularly among older subjects^47^. Due to other confounding factors, including sleep onset^10^ and sleep apnea^25^, any potential changes in sleep duration during follow-up remained undetected. A verified questionnaire scale of sleep quality is warranted for future studies, as these biases could lead to misclassification and underestimation of the association between sleep and bone density.

In summary, this analysis was based on a nationally representative sample using survey and inspection data. The results indicated that sleep duration < 5 h/day was associated with a higher risk of low bone density in women aged > 50 years with poor sleep quality. Our findings add to the current body of knowledge regarding relationships between bone health and the combined effect of sleep duration and gender. In future research, it is important to assess the potential causal effects of this association beyond the dimensions of the cross-sectional design.

## Supplementary Information

Supplementary Table S1.

Supplementary Table S2.

Supplementary Table S3.

## Data Availability

Large computerized datasets (NHIRD) were used to perform this nationwide population-based cohort study^48^.

## Abbreviations

BMD: Bone mineral density
CI: Confidence interval
DXA: Dual-energy X-ray absorptiometry
NHANES: National Health and Nutrition Examination Survey
OR: Odds ratio
PSQI: Pittsburgh Sleep Quality Index
