# Supplementary material for: A cross-sectional analysis of the association between sleep duration and osteoporosis risk in adults using 2005–2010 NHANES
Source: Sci Rep. 2021 Apr 27;11:9090. doi: 10.1038/s41598-021-88739-x (PMC8079413; doi:10.1038/s41598-021-88739-x)
Supplement: Supplementary file 1 — Supplementary Table S1. [file 41598_2021_88739_MOESM1_ESM.docx]

| Supplementary Table 1. Diagnosis of osteoporosis, osteopenia, or normal bone density based on T score over femoral trochanteric area. | | | | | | | | | | | |
| --- | --- | --- | --- | --- | --- | --- | --- | --- | --- | --- | --- |
|  | Sleeping hours per day | | | | | | | | | | P for trend |
|  | 1-4 | |  | 5-6 | |  | 7-8 |  | >9 | |  |
|  | OR 95%CI | P-Value |  | OR 95%CI | P-Value |  | OR 95%CI |  | OR 95%CI | P-Value |  |
| Overall |  |  |  |  |  |  |  |  |  |  |  |
| Osteoporosis vs. Normal | 4.587(2.206-9.539) | <.0001^＊＊^ |  | 1.732(1.117-2.687) | 0.0142 |  | REF |  | 1.81(0.993-3.299) | 0.0528 | 0.0143^＊^ |
| Low BMD vs. Normal | 1.582(1.238-2.021) | 0.0002^＊＊^ |  | 1.061(0.935-1.202) | 0.3586 |  | REF |  | 1.086(0.86-1.37) | 0.4879 | 0.0311^＊^ |
| Osteoporosis vs. Low BMD | 2.9(1.48-5.682) | 0.0019^＊^ |  | 1.633(1.038-2.57) | 0.0339 |  | REF |  | 1.667(0.947-2.934) | 0.0765 | 0.0482^＊^ |
| Male |  |  |  |  |  |  |  |  |  |  |  |
| Osteoporosis vs. Normal | 3.015(0.505-17.989) | 0.2259 |  | 1.321(0.468-3.73) | 0.599 |  | REF |  | 0.974(0.198-4.791) | 0.9741 | 0.2831 |
| Low BMD vs. Normal | 1.286(0.924-1.791) | 0.1363 |  | 0.991(0.809-1.214) | 0.9322 |  | REF |  | 1.1(0.766-1.581) | 0.6048 | 0.68 |
| Osteoporosis vs. Low BMD | 2.345(0.406-13.529) | 0.3407 |  | 1.333(0.472-3.766) | 0.5878 |  | REF |  | 0.885(0.199-3.933) | 0.8725 | 0.3095 |
| Female |  |  |  |  |  |  |  |  |  |  |  |
| Osteoporosis vs. Normal | 5.587(2.495-12.511) | <.0001^＊＊^ |  | 1.841(1.224-2.769) | 0.0034 |  | REF |  | 1.947(0.952-3.984) | 0.0682 | 0.0131^＊^ |
| Low BMD vs. Normal | 1.883(1.311-2.704) | 0.0006^＊＊^ |  | 1.117(0.958-1.303) | 0.1594 |  | REF |  | 1.098(0.763-1.579) | 0.6145 | 0.0199^＊^ |
| Osteoporosis vs. Low BMD | 2.968(1.417-6.214) | 0.0039^＊^ |  | 1.648(1.058-2.568) | 0.0271 |  | REF |  | 1.773(0.928-3.389) | 0.0829 | 0.0937 |
| Age<50 |  |  |  |  |  |  |  |  |  |  |  |
| Osteoporosis vs. Normal | 13.465(2.435-74.451) | 0.0029^＊＊^ |  | 2.027(0.477-8.618) | 0.3387 |  | REF |  | 7.765(1.643-36.702) | 0.0097 | 0.6755 |
| Low BMD vs. Normal | 1.343(0.969-1.861) | 0.0767 |  | 0.986(0.827-1.175) | 0.8746 |  | REF |  | 0.917(0.679-1.239) | 0.5732 | 0.3007 |
| Osteoporosis vs. Low BMD | 10.027(1.752-57.385) | 0.0096^＊^ |  | 2.056(0.469-9.003) | 0.3389 |  | REF |  | 8.467(1.876-38.205) | 0.0055 | 0.7452 |
| Age≥50 |  |  |  |  |  |  |  |  |  |  |  |
| Osteoporosis vs. Normal | 4.006(1.82-8.818) | 0.0006^＊＊^ |  | 1.769(1.14-2.746) | 0.011 |  | REF |  | 1.479(0.888-2.465) | 0.1331 | 0.0018^＊^ |
| Low BMD vs. Normal | 1.87(1.366-2.56) | <.0001^＊＊^ |  | 1.125(0.946-1.337) | 0.1831 |  | REF |  | 1.292(0.956-1.745) | 0.0956 | 0.0392^＊^ |
| Osteoporosis vs. Low BMD | 2.142(1-4.586) | 0.0499^＊^ |  | 1.573(0.979-2.527) | 0.0609 |  | REF |  | 1.145(0.662-1.981) | 0.6281 | 0.0135^＊^ |
| Sleep_disorder (-) |  |  |  |  |  |  |  |  |  |  |  |
| Osteoporosis vs. Normal | 3.505(1.065-11.534) | 0.0391 |  | 1.924(1.088-3.401) | 0.0244 |  | REF |  | 1.204(0.691-2.1) | 0.5121 | 0.0132^＊^ |
| Low BMD vs. Normal | 1.244(0.894-1.732) | 0.1958 |  | 0.998(0.864-1.151) | 0.9729 |  | REF |  | 1.016(0.776-1.331) | 0.9087 | 0.6354 |
| Osteoporosis vs. Low BMD | 2.817(0.837-9.485) | 0.0944 |  | 1.928(1.093-3.403) | 0.0235 |  | REF |  | 1.185(0.687-2.046) | 0.5412 | 0.0116 |
| Sleep_disorder (+) |  |  |  |  |  |  |  |  |  |  |  |
| Osteoporosis vs. Normal | 5.168(1.962-13.616) | 0.0009^＊＊^ |  | 1.54(0.841-2.821) | 0.1618 |  | REF |  | 3.946(1.158-13.445) | 0.0282 | 0.4266 |
| Low BMD vs. Normal | 1.597(1.091-2.34) | 0.0162^＊^ |  | 1.095(0.878-1.367) | 0.4213 |  | REF |  | 1.263(0.766-2.081) | 0.3602 | 0.1451 |
| Osteoporosis vs. Low BMD | 3.235(1.375-7.61) | 0.0071^＊^ |  | 1.406(0.787-2.513) | 0.2493 |  | REF |  | 3.125(1.013-9.644) | 0.0475 | 0.6873 |

^＊＊^ There is significant difference P <0.001.

^*^There is significant difference P<0.05.
