# Supplementary material for: A cross-sectional analysis of the association between sleep duration and osteoporosis risk in adults using 2005–2010 NHANES
Source: Sci Rep. 2021 Apr 27;11:9090. doi: 10.1038/s41598-021-88739-x (PMC8079413; doi:10.1038/s41598-021-88739-x)
Supplement: Supplementary file 2 — Supplementary Table S2. [file 41598_2021_88739_MOESM2_ESM.docx]

| Supplementary Table 2. Diagnosis of osteoporosis, low BMD, or normal bone density based on T score over femoral intertrochanteric area. | | | | | | | | | | | |
| --- | --- | --- | --- | --- | --- | --- | --- | --- | --- | --- | --- |
|  | Sleeping hours per day | | | | | | | | | | P for trend |
|  | 1-4 | |  | 5-6 | |  | 7-8 |  | >9 | |  |
|  | OR 95%CI | P-Value |  | OR 95%CI | P-Value |  | OR 95%CI |  | OR 95%CI | P-Value |  |
| Overall |  |  |  |  |  |  |  |  |  |  |  |
| Osteoporosis vs. Normal | 0.994(0.379-2.609) | 0.9908 |  | 1.503(0.965-2.339) | 0.0714 |  | REF |  | 1.557(0.837-2.894) | 0.162 | 0.5713 |
| Low BMD vs. Normal | 1.209(0.924-1.582) | 0.1669 |  | 1.08(0.963-1.211) | 0.1886 |  | REF |  | 1.435(1.091-1.889) | 0.0099 | 0.8149 |
| Osteoporosis vs. Low BMD | 0.822(0.339-1.995) | 0.6656 |  | 1.392(0.931-2.08) | 0.1071 |  | REF |  | 1.084(0.597-1.969) | 0.7899 | 0.4617 |
| Male |  |  |  |  |  |  |  |  |  |  |  |
| Osteoporosis vs. Normal | 0.822(0.2-3.376) | 0.7854 |  | 0.853(0.305-2.384) | 0.7618 |  | REF |  | 0.463(0.111-1.932) | 0.2905 | 0.8824 |
| Low BMD vs. Normal | 1(0.604-1.654) | 0.9992 |  | 1.096(0.914-1.313) | 0.3224 |  | REF |  | 1.587(1.073-2.347) | 0.0206 | 0.5219 |
| Osteoporosis vs. Low BMD | 0.822(0.181-3.728) | 0.7994 |  | 0.779(0.273-2.216) | 0.6391 |  | REF |  | 0.292(0.067-1.266) | 0.1 | 0.7422 |
| Female |  |  |  |  |  |  |  |  |  |  |  |
| Osteoporosis vs. Normal | 1.076(0.345-3.356) | 0.8998 |  | 1.688(1.013-2.811) | 0.0443^＊＊^ |  | REF |  | 1.806(0.876-3.723) | 0.1095 | 0.5927 |
| Low BMD vs. Normal | 1.4(0.922-2.126) | 0.1139 |  | 1.062(0.887-1.272) | 0.512 |  | REF |  | 1.364(0.954-1.949) | 0.0884 | 0.8541 |
| Osteoporosis vs. Low BMD | 0.768(0.293-2.012) | 0.5915 |  | 1.589(1.015-2.487) | 0.0428 |  | REF |  | 1.324(0.699-2.508) | 0.3892 | 0.5608 |
| Age<50 |  |  |  |  |  |  |  |  |  |  |  |
| Osteoporosis vs. Normal | No event |  |  | 4.866(1.17-20.233) | 0.0295^＊^ |  | REF |  | 10.149(1.387-74.241) | 0.0225 | 0.8125 |
| Low BMD vs. Normal | 0.808(0.487-1.338) | 0.4068 |  | 0.953(0.764-1.189) | 0.6693 |  | REF |  | 1.21(0.761-1.924) | 0.4197 | 0.2425 |
| Osteoporosis vs. Low BMD | No event |  |  | 5.106(1.21-21.557) | 0.0265^＊^ |  | REF |  | 8.386(1.169-60.174) | 0.0344 | 0.9012 |
| Age≥50 |  |  |  |  |  |  |  |  |  |  |  |
| Osteoporosis vs. Normal | 1.26(0.455-3.491) | 0.6563 |  | 1.447(0.896-2.337) | 0.1306 |  | REF |  | 1.397(0.768-2.543) | 0.2735 | 0.2748 |
| Low BMD vs. Normal | 1.633(1.181-2.258) | 0.003^＊＊^ |  | 1.17(0.981-1.394) | 0.08^＊^ |  | REF |  | 1.659(1.26-2.184) | 0.0003 | 0.5142 |
| Osteoporosis vs. Low BMD | 0.772(0.317-1.878) | 0.5681 |  | 1.237(0.795-1.927) | 0.3459 |  | REF |  | 0.842(0.468-1.517) | 0.568 | 0.3735 |
| Sleep_disorder (-) |  |  |  |  |  |  |  |  |  |  |  |
| Osteoporosis vs. Normal | 0.637(0.149-2.724) | 0.5426 |  | 1.78(1.136-2.787) | 0.0118 |  | REF |  | 1.395(0.806-2.415) | 0.2346 | 0.1798 |
| Low BMD vs. Normal | 0.924(0.595-1.434) | 0.7235 |  | 1.108(0.953-1.287) | 0.1816 |  | REF |  | 1.563(1.178-2.074) | 0.002 | 0.2571 |
| Osteoporosis vs. Low BMD | 0.689(0.162-2.937) | 0.6146 |  | 1.607(1.068-2.417) | 0.0229^＊^ |  | REF |  | 0.892(0.507-1.571) | 0.6931 | 0.0414^＊^ |
| Sleep_disorder (+) |  |  |  |  |  |  |  |  |  |  |  |
| Osteoporosis vs. Normal | 0.854(0.205-3.57) | 0.8293 |  | 0.974(0.407-2.334) | 0.9531 |  | REF |  | 1.762(0.426-7.291) | 0.4345 | 0.4869 |
| Low BMD vs. Normal | 1.181(0.775-1.798) | 0.4387 |  | 0.956(0.72-1.271) | 0.7588 |  | REF |  | 1.133(0.632-2.033) | 0.6747 | 0.9196 |
| Osteoporosis vs. Low BMD | 0.724(0.229-2.284) | 0.5813 |  | 1.018(0.457-2.269) | 0.9643 |  | REF |  | 1.555(0.428-5.647) | 0.5027 | 0.4334 |

^＊＊^ There is significant difference P <0.001.

^*^There is significant difference P<0.05.
