# Supplementary material for: A cross-sectional analysis of the association between sleep duration and osteoporosis risk in adults using 2005–2010 NHANES
Source: Sci Rep. 2021 Apr 27;11:9090. doi: 10.1038/s41598-021-88739-x (PMC8079413; doi:10.1038/s41598-021-88739-x)
Supplement: Supplementary file 3 — Supplementary Table S3. [file 41598_2021_88739_MOESM3_ESM.docx]

| Supplementary Table 3. Diagnosis of osteoporosis, osteopenia, or normal bone density based on T score over total proximal femoral area. | | | | | | | | | | | |
| --- | --- | --- | --- | --- | --- | --- | --- | --- | --- | --- | --- |
|  | Sleeping hours per day | | | | | | | | | | P for trend |
|  | 1-4 | |  | 5-6 | |  | 7-8 |  | >9 | |  |
|  | OR 95%CI | P-Value |  | OR 95%CI | P-Value |  | OR 95%CI |  | OR 95%CI | P-Value |  |
| Overall |  |  |  |  |  |  |  |  |  |  |  |
| Osteoporosis vs. Normal | 1.33(0.624-2.835) | 0.4608 |  | 1.488(0.997-2.222) | 0.0517 |  | REF |  | 1.7(0.97-2.98) | 0.0639 | 0.5251 |
| Low BMD vs. Normal | 1.394(1.091-1.78) | 0.0078^＊^ |  | 1.103(0.975-1.248) | 0.1188 |  | REF |  | 1.32(1.006-1.733) | 0.045 | 0.2989 |
| Osteoporosis vs. Low BMD | 0.954(0.478-1.903) | 0.8942 |  | 1.349(0.938-1.94) | 0.1065 |  | REF |  | 1.287(0.764-2.168) | 0.3421 | 0.7431 |
| Male |  |  |  |  |  |  |  |  |  |  |  |
| Osteoporosis vs. Normal | 0.677(0.177-2.586) | 0.5685 |  | 0.71(0.297-1.694) | 0.4401 |  | REF |  | 0.545(0.152-1.963) | 0.3535 | 0.7135 |
| Low BMD vs. Normal | 1.089(0.73-1.623) | 0.6764 |  | 1.087(0.904-1.306) | 0.3737 |  | REF |  | 1.482(1.029-2.132) | 0.0344 | 0.7467 |
| Osteoporosis vs. Low BMD | 0.622(0.159-2.435) | 0.4952 |  | 0.653(0.277-1.539) | 0.3299 |  | REF |  | 0.368(0.102-1.324) | 0.1259 | 0.7871 |
| Female |  |  |  |  |  |  |  |  |  |  |  |
| Osteoporosis vs. Normal | 1.775(0.683-4.616) | 0.239 |  | 1.778(1.136-2.782) | 0.0118 |  | REF |  | 2.025(1.011-4.056) | 0.0464 | 0.3965 |
| Low BMD vs. Normal | 1.725(1.119-2.66) | 0.0135^＊^ |  | 1.116(0.921-1.351) | 0.262 |  | REF |  | 1.244(0.849-1.822) | 0.2621 | 0.1907 |
| Osteoporosis vs. Low BMD | 1.029(0.462-2.291) | 0.9443 |  | 1.593(1.065-2.382) | 0.0233 |  | REF |  | 1.628(0.896-2.957) | 0.1094 | 0.7538 |
| Age<50 |  |  |  |  |  |  |  |  |  |  |  |
| Osteoporosis vs. Normal | No event |  |  | 2.423(0.704-8.346) | 0.1606 |  | REF |  | 7.608(1.471-39.347) | 0.0155 | 0.4731 |
| Low BMD vs. Normal | 1.127(0.795-1.596) | 0.5025 |  | 1.018(0.822-1.261) | 0.8686 |  | REF |  | 1.158(0.787-1.703) | 0.4574 | 0.9845 |
| Osteoporosis vs. Low BMD | No event |  |  | 2.38(0.658-8.605) | 0.1861 |  | REF |  | 6.572(1.323-32.641) | 0.0213 | 0.4745 |
| Age ≥50 |  |  |  |  |  |  |  |  |  |  |  |
| Osteoporosis vs. Normal | 1.676(0.734-3.828) | 0.2203 |  | 1.463(0.937-2.282) | 0.0941 |  | REF |  | 1.472(0.938-2.31) | 0.0927 | 0.1912 |
| Low BMD vs. Normal | 1.699(1.182-2.443) | 0.0042^＊^ |  | 1.166(0.986-1.38) | 0.0723 |  | REF |  | 1.49(1.103-2.014) | 0.0094 | 0.2426 |
| Osteoporosis vs. Low BMD | 0.987(0.486-2.001) | 0.9701 |  | 1.254(0.847-1.857) | 0.2586 |  | REF |  | 0.988(0.621-1.57) | 0.9581 | 0.4045 |
| Sleep disorder (-) |  |  |  |  |  |  |  |  |  |  |  |
| Osteoporosis vs. Normal | 0.91(0.423-1.96) | 0.8105 |  | 1.664(1.066-2.597) | 0.0249 |  | REF |  | 1.299(0.806-2.094) | 0.2825 | 0.1368 |
| Low BMD vs. Normal | 1.127(0.781-1.626) | 0.5231 |  | 1.123(0.975-1.295) | 0.1081 |  | REF |  | 1.38(1.04-1.83) | 0.0255 | 0.9867 |
| Osteoporosis vs. Low BMD | 0.808(0.39-1.674) | 0.5661 |  | 1.481(0.992-2.211) | 0.0545 |  | REF |  | 0.942(0.578-1.534) | 0.8092 | 0.0851 |
| Sleep disorder (+) |  |  |  |  |  |  |  |  |  |  |  |
| Osteoporosis vs. Normal | 1.313(0.376-4.582) | 0.6693 |  | 1.18(0.541-2.574) | 0.6782 |  | REF |  | 2.762(0.76-10.043) | 0.1229 | 0.5121 |
| Low BMD vs. Normal | 1.349(0.919-1.98) | 0.1268 |  | 0.977(0.746-1.28) | 0.8677 |  | REF |  | 1.136(0.623-2.071) | 0.6784 | 0.5146 |
| Osteoporosis vs. Low BMD | 0.974(0.341-2.78) | 0.9601 |  | 1.207(0.608-2.396) | 0.5909 |  | REF |  | 2.433(0.798-7.415) | 0.118 | 0.3431 |

^＊＊^ There is significant difference P <0.001.

^*^ There is significant difference P<0.05.
